# Zirconium Phosphate Assisted Phosphoric Acid Co-Catalyzed Hydrolysis of Lignocellulose for Enhanced Extraction of Nanocellulose

**DOI:** 10.3390/polym15020447

**Published:** 2023-01-14

**Authors:** Hanchen Wang, Jiayin Wu, Yuan Lian, Yonggui Li, Biao Huang, Qilin Lu

**Affiliations:** 1 College of Material Engineering, Fujian Agriculture and Forestry University, Fuzhou 350002, China; 2 Fujian Key Laboratory of Novel Functional Textile Fibers and Materials, Minjiang University, Fuzhou 350108, China

**Keywords:** zirconium phosphate, acid hydrolysis, CNCs, optimization, response surface methodology

## Abstract

The high mechanical strength, large specific surface area, favorable biocompatibility, and degradability of nanocellulose (CNC) enable it to be a potential alternative to petroleum-based materials. However, the traditional preparation of CNCs requires a large amount of strong acid, which poses a serious challenge to equipment maintenance, waste liquid recycling, and economics. In this study, a solid and easily recoverable zirconium phosphate (ZrP) was used to assist in the phosphoric acid co-catalyzed hydrolysis of lignocellulose for extracting CNCs. Due to the presence of acidic phosphate groups, ZrP has a strong active center with a high catalytic activity. With the assistance of ZrP, the amount of phosphoric acid used in the reaction is significantly reduced, improving the equipment’s durability and economic efficiency. The effects of the process conditions investigated were the phosphate acid concentration, reaction temperature, and reaction time on the yield of CNCs. The Box–Behnken design (BBD) method from the response surface methodology (RSM) was applied to investigate and optimize the preparation conditions. The optimized pre-treatment conditions were 49.27% phosphoric acid concentration, 65.38 °C reaction temperature, and 5 h reaction time with a maximal cellulose yield (48.33%). The obtained CNCs show a granular shape with a length of 40~50 nm and a diameter of 20~30 nm, while its high zeta potential (−24.5 mV) make CNCs present a stable dispersion in aqueous media. Moreover, CNCs have a high crystallinity of 78.70% within the crystal type of cellulose Ⅰ. As such, this study may pioneer the horizon for developing a green method for the efficient preparation of CNC, and it is of great significance for CNCs practical production process.

## 1. Introduction

The abuse of petroleum-based materials has not only resulted in a rapid decline in their storage capacity but has also led to a number of serious ecological problems [1,2,3]. Growing societal concern for the earth’s ecology, sustainability concepts, and rigorous government regulations have conspired to worry about our dependence on non-renewable petroleum-based materials and stimulated the exploration of novel and environmentally friendly materials and processes [4]. Recently, nanocellulose (CNCs) has become a potential alternative to petroleum-based materials by virtue of its high strength, high specific surface area, excellent biocompatibility, degradability, and renewable properties [5,6,7]. CNCs can be extracted from various plant resources, commonly used such as microcrystalline cellulose (MCC), wood pulp, cotton, hemp, bacterial cellulose, and crop waste, which was prepared by different raw materials and different methods usually have large differences in their morphology, crystallinity, and dimensions [8,9,10]. Pennisetum Sinese Roxb (PSR) is a high-yielding and high-quality mycorrhiza suitable for growth and artificial cultivation in tropical, subtropical, and temperate zones [11]. Owing to its capacity for a rapid biomass accumulation, PSR is an ideal cellulosic source for producing biomaterials and biofuels, as well as a significant source of high-quality fibers as a substitute for wood and synthetic fibers or fillers. Cellulose accounts for about 25–30% of the chemical composition of PSR. The high-quality and abundant cellulose content makes it ideal for the preparation of CNCs.

The conventional chemical preparation of CNCs is to use strong acid to hydrolysis the β-1,4 glycosidic bond between cellulose molecules, which is a kind of acetal bond that is easily hydrolyzed in the presence of strong acids [12,13]. The hydrogen ions ionized by the strong acid are first transferred to the interior of the cellulose to destroy and degrade the amorphous region in the cellulose molecule and then penetrate into the part of the defective crystalline region to degrade it. Finally, the crystalline region of the cellulose is retained to obtain CNCs [14]. However, this preparation method will produce a large amount of waste liquid that is difficult to recycle, seriously corrode production equipment, and disposing of them will cause a serious pollution to the environment. Compared with inorganic acids, solid acids have the advantages of being reusable, less corrosive to equipment, and less polluting to the environment, replacing inorganic acids with solid acids is a compelling research direction in green chemistry [15,16,17]. As a solid acid, zirconium phosphate (ZrP) has a strong catalytic activity due to the presence of phosphate groups within its structure which increases the number of acidic sites of the catalyst accessible to the reactants. The acidic phosphate group forms a powerful active center and releases a large number of hydrogen protons, which can greatly reduce the amount of phosphoric acid used in the acid hydrolysis process and reduce the generation of waste solution. In addition, ZrP is characterized by a large thermal stability and mechanical strength and are practically insoluble in water, which means it can be easily recycled and reused [18]. To our knowledge, ZrP-assisted phosphoric acid co-catalyzed hydrolysis for the preparation of CNCs from PSR has not been investigated before.

Response surface methodology (RSM) uses multiple quadratic regression equations to analyze the relationship between the factors and experimental results to obtain better process parameters. This method is widely used in manufacturing, agriculture, medicine, and the chemical industry because of its high accuracy and low number of tests [19]. In this study, the treatment of ZrP-assisted phosphoric acid co-catalyzed hydrolysis PSR to prepare CNCs was measured on the yield of CNCs to estimate the feasibility of the process based on the CNCs extraction. The optimization of the treatment conditions (phosphate concentration, reaction temperature, and reaction time) was achieved statistically by response surface methodology. Changes in the morphology and characteristics of cellulose imparted by the treatment were characterized through a fiber analyzer, transmission electron microscope (TEM), Fourier Transform Infrared Spectrometer (FT-IR), X-ray diffraction (XRD), ZETA potentiometry, and thermal analyses. This study blazes the trail to utilize the low-cost and readily available PSR for CNCs production through an optimized ZrP-assisted phosphoric acid co-catalyzed hydrolysis process.

## 2. Materials and Methods

### 2.1. Materials

PSR was obtained from the China National Engineering Research Center of JUNCAO Technology (Fuzhou, China); phosphoric acid (AR) and sodium hydroxide (AR) were purchased from Sinopharm Chemical Reagent Co., Ltd. (Shanghai, China); zirconium phosphate (AR) was purchased from Xiamen Xindakang Inorganic Material Co., Ltd. (Xiamen, China).

### 2.2. Extraction of Cellulose

The treatment of raw PSR by sodium hydroxide to prepare cellulose included the following conditions: 30 g of dried PSR was heated at 165 °C in 180 mL of 18 wt.% NaOH for 2 h to remove most of the lignin. After that, the insoluble residue was filtered and washed with deionized water, the remained lignin was disposed by a 7% NaOCl solution treatment, then the obtained cellulose was repeatedly filtered and washed with deionized water until the pH of the filtrate was neutral. At last, the obtained PSR-cellulose was dried at 50 °C for 24 h.

### 2.3. Production of CNCs

A total of 2 g of cellulose and various amounts of ZrP (0.1, 0.5, 1.0, and 1.5 g) were placed in a 100 mL phosphoric acid solution, then the mixture was ultrasonicated with a frequency of 40 kHz at the ultrasonic power of 250 W. After that, the ZrP was separated. The CNCs were purified by centrifugation at 10,000 rpm for 10 min, then the upper suspension was collected to obtain the CNCs colloid and freeze-dried to obtain CNCs powder.

### 2.4. Optimization of Preparation of CNCs Conditions

The Box–Behnken Design (BBD) of response surface methodology was applied to design a suite of experiments for the optimization of the effective parameters in the preparation of CNCs by ZrP-assisted phosphoric acid co-catalytic hydrolysis of PSR. A quadratic model with 3 factors and 17 experiments was employed, including 5 replications to estimate the error. The design variables of 3 factors were the phosphate concentration (A), reaction temperature (B), and reaction time (C), while the response variables were the CNCs yield. As illustrated in Table 1, each factor had three levels, low, mid, and high, denominated as −1, 0, and 1, respectively.

### 2.5. Characterization

#### 2.5.1. The Yield of CNCs

A total of 25 mL of the CNCs colloid collected in Section 2.3 was placed into in a weighing bottle, freeze-dried for 48 h, and then weighed. The yield of the CNCs (%) was calculated by Equation (1):

(1) $$Y\left(\%\right)=\frac{\left(m_{1}-m_{2}\right)V}{25m}\times 100$$

where *m*_1_ is the dry weight of the total CNCs and weighing bottle (g), *m*_2_ is the weight of the weighing bottle, *m* is the dry weight of the starting material (g), and *V* is the total volume of the CNCs colloid (mL) collected in Section 2.3. 

#### 2.5.2. Fiber Morphology Analysis

A fiber analyzer (Morfi Compact, Techpap Co., Ltd., Lyon, France) was applied to explore the effect of the dissociation process on the PSR morphology, 0.02 g of dried PSR fiber and dissociated PSR fiber was diluted by deionized water as 0.02 g/L of suspension, and it was characterized by a fiber analyzer at room temperature.

#### 2.5.3. Morphological Characterization by TEM

Aqueous CNCs and PSR fiber suspensions of a 0.1% (*w*/*v*) concentration were sonicated for 20 min and the droplet was placed on a carbon-coated copper grid. After drying, the sample was negatively stained with 2% phosphotungstic acid dye for 60 s and left to dry at room temperature. Then, the transmission electron microscope (TEM) (Hitachi-H7650, Hitachi, Ltd., Tokyo, Japan) was operated at 100 kV to measure each sample morphology.

#### 2.5.4. Fourier Transform Infrared Spectroscopy (FTIR)

The CNCs and PSR-cellulose were dried, grounded, pelletized using KBr and scanned by an FTIR spectrophotometer (Thermo Electron Instruments Co., Ltd., Madison, WI, USA). Each spectrum was acquired by averaging 32 scans per sample in the mid-infrared range (500–4000 cm^−1^) at a 4 cm^−1^ spectral resolution.

#### 2.5.5. X-ray Diffraction (XRD)

An X-Ray Powder Diffractometer (X’Pert Pro MPD, Philips-FEI, Amsterdam, The Netherlands) was used to obtain the XRD spectra. The Cu-Kα scattering radiation is detected at a scanning rate of 0.1°/s in the range of 2θ = 6~90° at 50 kV and 300 mA. The degree of crystallinity (Crl, %) was calculated by Equation (2) [20]:

(2) $$\mathrm{CrI}=\left(I_{002}-I_{\mathrm{am}}\right)/I_{002}$$

where I_002_ is the maximum intensity of the (002) diffraction at 2θ value of about 22.2°, while I_am_ is the intensity diffraction at 2θ value of around 18°.

#### 2.5.6. Zeta Potential Analysis

A system ZETA potentiometry instrument (SZP-06, BTG Co., Ltd., Almholt, Switzerland) was used to determine the surface charge of PSR and CNCs. The change of the ZETA potential values was contributed to know CNCs dispersion stability in the aqueous. A total of 0.02 g of sample was diluted by deionized water as a 1 g/L suspension, which was fully sonicated and characterized at room temperature.

#### 2.5.7. Thermal Gravimetric Analysis (TGA)

The thermogravimetric stability of CNCs and PSR cellulose was analyzed using a thermal analyzer (STA449F3 thermal analyzer, NETZSCH Co., Ltd., Munich, Germany). The TGA analysis was performed in a 150 mL/min N_2_ flow with a heating and cooling rate of 10 °C/min within 25–600 °C.

## 3. Results and Discussion

### 3.1. Chemical Composition of PSR

The PSR chemical composition varies according to the growth time, and the nitrate ethanol method was used to determine the main chemical composition of PSR. The main components of PSR in different production stages are shown in Table 2. As seen in Table 2, the PSR with a growth cycle of 8 weeks had the least ash content and a high fiber content similar to the ten weeks, so the PSR with a growth cycle of 8 weeks was selected to extract CNCs.

### 3.2. Optimization of CNCs Preparation Conditions

#### 3.2.1. Response Surface Analysis

The levels of each factor for the 17 experiments designed according to the RSM design are shown in Table 3, and the corresponding CNCs yields for each round are presented in parallel. The yields of CNCs prepared from PSR fibers varied between 40.00 and 49.00% under different conditions.

The analysis of variance (ANOVA) method was adopted to evaluate the significance level and accuracy of the fitted model (Table 2). If the value of “Prob > F” is less than 0.05 and 0.0001, respectively, it means that the effect of the model term is significant and highly significant, respectively. Another condition that proves that the model’s compatibility is significant is that the F value is greater than or equal to six [21]. The F value of the quadratic polynomial model is 67.60, and the “Prob > F” is less than 0.0001, meaning that the model is highly significant. The value of “Prob > F” for the Lack of Fit term is 0.8039, which is much larger than 0.1000, indicating that the Lack of Fit term of the model is not significant, suggesting that the quadratic polynomial model suggested that the model described the CNCs preparation data well. Moreover, the coefficient of determination R^2^ and the R^2^ correction value of the model can reflect the degree of model fit. The R^2^ and Adj R^2^ of the quadratic polynomial model is 0.9886 and 0.9740, which indicates that the correlation between the predicted value and the test value of the model reaches 98.86% and the model can reflect 97.40% of the variation in the response value, respectively [22]. The result reveals that the experiments designed with a quadratic polynomial model have less errors and can analyze and predict the preparation of CNCs accurately. The relationship between the CNCs yield (%) and the independent variables is given by the regression Equation (3).

Y = 48.17 − 1.05A + 0.49B − 0.74C + 0.083AB − 0.085AC + 0.0009BC − 3.54A^2^ − 3.12B^2^ − 1.87C^2^ (3)

The values of F and Prob > F in ANOVA (Table 4) indicated that the linear terms (A, B, and C) and quadratic terms (A^2^, B^2^ and C^2^) have a significant effect on the CNCs yield, however, the interaction terms (AB, AC, and BC) have less significant effects on the response values, indicating that the phosphoric acid concentration, reaction temperature, and reaction time all had significant effects on the yield of CNCs, while the effects of the interactions between the factors were less significant. The three factors influenced the response values in the following order: phosphoric acid concentration > reaction time > reaction temperature.

#### 3.2.2. Surface Plots, Optimization and Model Verification

**Figure 1:**
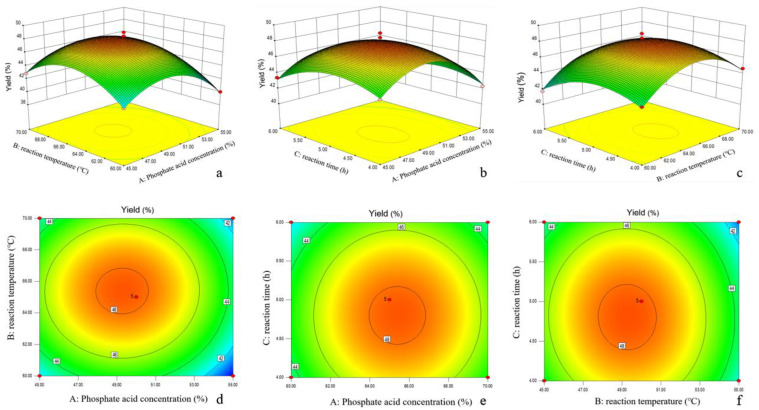
Three-dimensional response surface plots (**a**–**c**) and corresponding contour plots (**d**–**f**) of the effect of phosphate acid concentration, reaction temperature, and reaction time on CNCs Yield.

The constructed 3D response surface plots and corresponding contour plots are shown in Figure 1. These plots depict the synergistic effects of the two factors on the yield of CNCs, while the unobserved factors remain constant at their medium levels. The effect of the two-factor interaction on the CNCs yield can be judged by the shape of the contour plots, with elliptical contour plots indicating significant interactions between variables and circular contour plots indicating those with insignificant interactions [23]. However, only the interaction between the acid concentration and the temperature was significant. As is shown in Figure 1, the three independent variables of the phosphoric acid concentration, reaction temperature, and reaction time all had significant effects on the yield of the CNCs. However, only the interaction of the reaction temperature and reaction time was significant (Figure 1f).

When the other variables were kept at a medium level, the yield of the CNCs increased gradually with the phosphoric acid concentration increasing until 50% and then decreasing, which could be owing to the fact that phosphoric acid can promote the release of hydrogen protons from zirconium phosphate, and the hydrogen protons in turn to act on the breakage of the β-1,4 glycosidic bond within the cellulose molecule, decreasing the cellulose polymerization and hydrolyzing the amorphous region to obtain CNCs. On the other hand, excess phosphoric acid will result in the excessive hydrolysis of fibers to glucose, leading to the decrease in the CNCs yield. Meanwhile, the effect of the reaction temperature conditions and reaction time conditions on the cellulose yield showed a similar trend to that of the phosphoric acid concentration. The yield of the CNCs reached the maximum at the reaction temperature of 65 °C and the reaction time of 5 h, respectively. A high reaction temperature or long reaction time can lead to the excessive hydrolysis of CNCs, resulting in lower yields. Furthermore, when the reaction temperature and time levels were at high levels, the CNCs yields remained low in spite of the phosphoric acid concentration, thus demonstrating the importance of reaction temperature × reaction time interactions for maximizing the CNCs yields.

The optimum conditions for the CNCs preparation from PSR with the highest yield were identified using Design-Expert. The predicted optimal reaction conditions were an acid concentration of 49.27%, a temperature of 65.38 °C, a time of 5 h, and the prediction yield was 48.33%, which were coherent with the experimental results. CNCs were prepared from PSR with a yield of 50.00%, using a phosphoric acid concentration of 49% at 65 °C for 5 h. This is in close agreement with the model’s results, i.e., within the 95% confidence interval, thus validating the sufficiency and accuracy of the model [24].

### 3.3. Morphology Analysis

**Figure 2:**
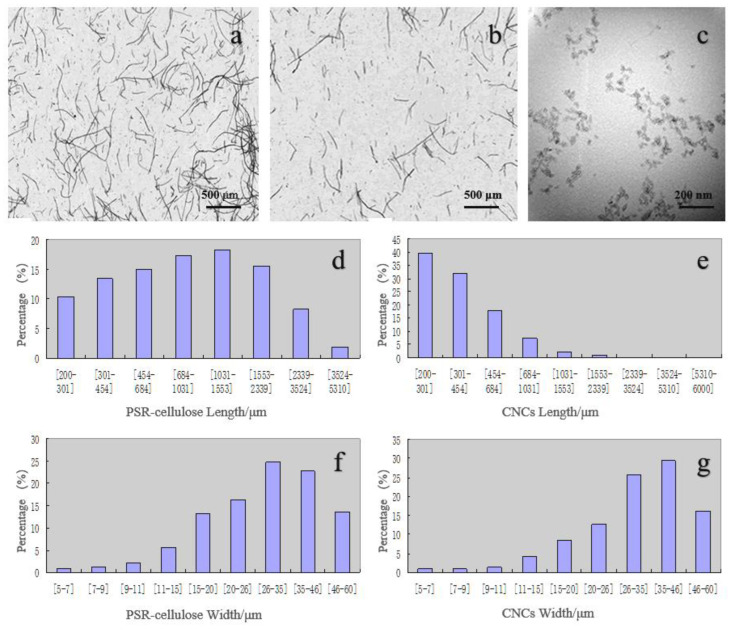
Fiber analyzer micrograph of PSR-cellulose (**a**) and CNCs (**b**); TEM micrograph of CNCs (**c**); length distribution chart of PSR-cellulose (**d**) and CNCs (**e**); width distribution chart of PSR-cellulose (**f**) and CNCs (**g**).

The fiber analyzer was used to investigate the changes in morphology and microstructure of PSR-cellulose and CNCs. As is depicted in Figure 2a,b, both PSR-cellulose and CNCs can be observed with a relatively large L/W ratio and a similar rod-like shape. Figure 2d,e show the length distribution of PSR-cellulose and CNCs calculated by the fiber analyzer, respectively. The length of PSR-cellulose was mainly distributed between 200 and 3524 μm, while the length of CNCs was mainly distributed between 200 and 1031 μm, and 45% of the PSR fibers is longer than 1000 μm, which decreases to 5% after hydrolysis. On the other hand, the main distribution range of the CNCs width was between 20 and 60 μm, which is more concentrated than PSR-cellulose (Figure 2f,g). This indicates that phosphoric acid hydrolysis leads to the decomposition of the cellulose structure, the breakage of PSR-cellulose chains, and the CNCs were obtained under the assistance of ZrP. 

To further observe the morphology of CNCs, TEM was conducted (Figure 2c). In the TEM views, the CNCs obtained by ZrP-assisted phosphoric acid co-catalyzed hydrolysis PSR present as the granular form. The average size of the CNCs was measured, and the diameter of the CNCs produced from the PSR was found to be approximately 20~30 nm, while the average length was 40~50 nm. The TEM observations indicate that the cellulose obtained from ZrP-assisted phosphoric acid co-catalyzed hydrolysis PSR-cellulose is of a nanoscale. 

### 3.4. FTIR Analysis

**Figure 3:**
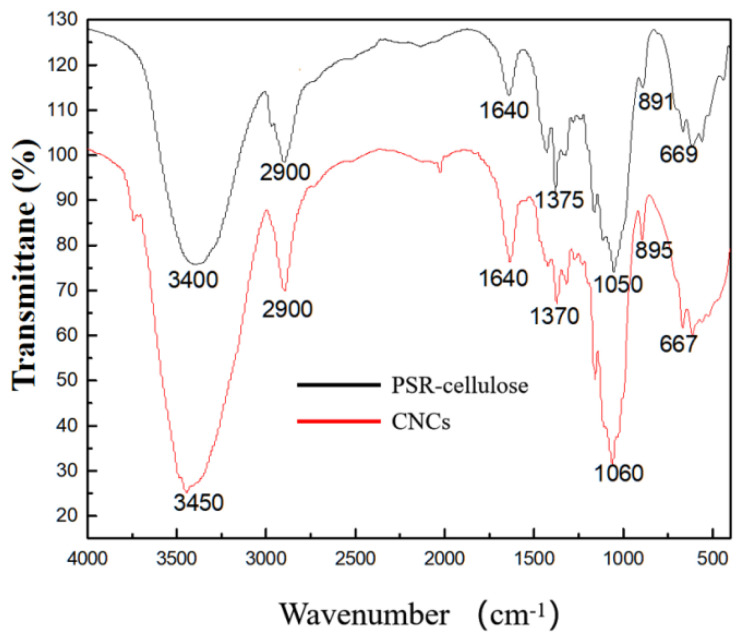
FTIR spectra of PSR-cellulose and CNCs.

The FTIR spectra of PSR-cellulose and CNCs presented similar spectra in Figure 3, and the characteristic peaks of CNCs were almost not shifted after hydrolysis, indicating that the chemical structure of the CNCs was not destroyed or changed, and the basic backbone structure of PSR-cellulose was still maintained [25]. At a high wavenumber, a broad peak caused by the aliphatic and phenolic O-H stretching vibrations of cellulose is positioned at 3450 cm^−1^, while another absorption peak formed by the deformation and stretching vibrations of the C-H and OH-groups of the glucose unit is found at 2900 cm^−1^ [26]. Further, the peaks nearby 1060 cm^−1^ and 895 cm^−1^ are both from the backbone of the cellulose chain, which can be attributed to the CO-stretching of secondary alcohols and ether groups and β-1,4-glycoside-linked O-H stretching, respectively [27]. The absorbed water of PSR-cellulose and CNCs is observed at 1640 cm^−1^ [28].

### 3.5. Crystal Structure

**Figure 4:**
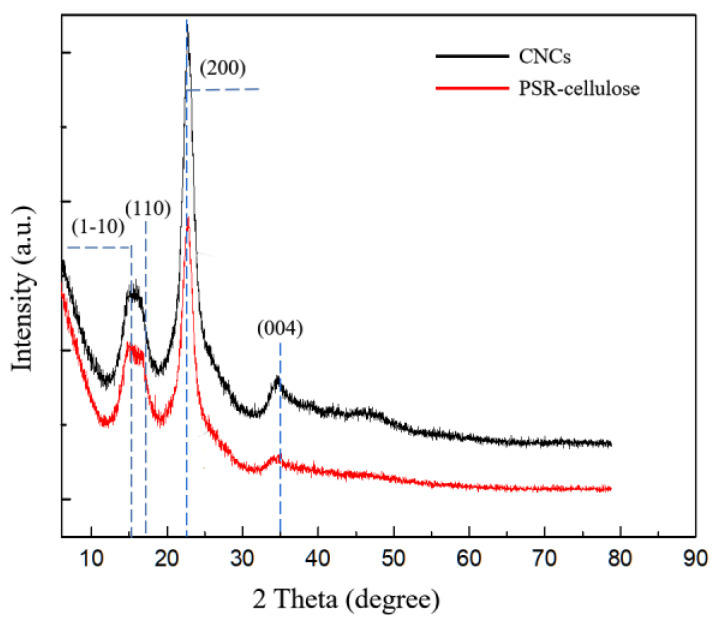
XRD spectra of PSR-cellulose and CNCs.

The XRD spectra of the PSR-cellulose and CNCs are depicted in Figure 4. All samples exhibit four diffraction peaks nearby 2θ = 15.5°, 17.5°, 23°, and 35°, corresponding to (1–10), (110), (200), and (400) diffraction planes of cellulose lattice, respectively, suggesting that the crystalline type of CNCs is not altered in the manufacture processing and the CNCs remain the cellulose I crystal form [29,30]. Crystalline cellulose exists in four isomeric forms as I–IV, of which cellulose I is the most common crystalline cellulose of a natural origin [31].

The Crl values for the PSR-cellulose and CNCs were 68.12 and 78.70%, respectively. As the alkali and bleaching treatment steps resulted in the removal of amorphous hemicellulose and lignin from the region, the crystallinity of PSR-cellulose was high (68.12%). After the ZrP-assisted phosphoric acid co-catalyzed hydrolysis PSR, the crystallinity of the CNCs raised to 78.70%, indicating that the amorphous region and parts of the defective crystalline regions in PSR-cellulose were affected by phosphoric acid hydrolysis catalyzing by zirconium phosphate. During this process, hydrogen ions enter into the amorphous region of cellulose to accelerate the hydrolytic splitting of the glycosidic bonds, and the amorphous region of cellulose and the surface of the crystalline region are partially destroyed, thus making the CNCs crystallinity higher than that of natural cellulose. On the other hand, some cellulose single crystals undergo a rearrangement, which further increases the CNCs crystallinity.

### 3.6. Zeta Potentiometric Analysis

The PSR and the CNCs under different preparation conditions were measured by the ZETA potentiostat, and the results are shown in Table 5. The zeta potential represents the effective charge of the charged particles dispersed in a liquid-phase medium. A higher absolute value of potential means a stronger mutual repulsion between particles and a better stability of the dispersed system cellulose fibers are negatively charged in aqueous media owing to the structure of cellulose-containing glyoxylate groups, polar hydroxyl groups, etc. Compared to PSR, the zeta potential values of CNCs were significantly higher, indicating that CNCs have a good dispersion in aqueous media. This is mainly because the lateral repulsion generated by PO_4_^3−^ during the preparation of ZrP-assisted catalytic phosphate hydrolysis resulted in a stronger repulsion between the particles and increased zeta potential values.

### 3.7. Thermal Analysis

**Figure 5:**
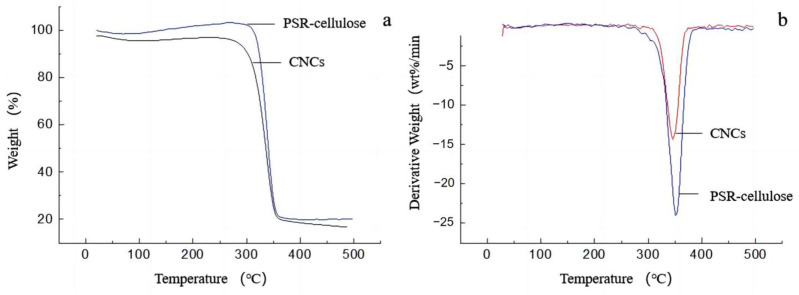
(**a**) TG and (**b**) DTG curves of PSR-cellulose and CNCs.

The TG and DTG profiles of PSR-cellulose and CNCs are shown in Figure 5. As Figure 5a depicted, the weight loss below 120 °C for two samples can be attributed to the water evaporation [32], the main thermal decomposition temperature of the molecular structure ranges from 310 °C to 380 °C, and the carbonation occurs when the temperature rises above 400 °C. The calculated onset decomposition temperature of PSR-cellulose and CNCs is 321.8 °C and 313.5 °C, and the maximum decomposition temperature is 351.0 °C and 330.4 °C, respectively (Figure 5b). It has been reported that the thermal stability of CNCs is enhanced thanks to the removal of the amorphous region from the cellulose and the rearrangement of the crystal sequence in the crystalline region [33], however, the thermal analysis results showed that the thermal onset decomposition temperature of CNCs was decreased compared to PSR-cellulose. This may be due to the fact that although most of the lignin and hemicellulose were removed after the alkali bleaching treatment of PSR-cellulose, there was a strong linkage between some of the lignin and cellulose, forming a lignin–cellulose complex, and thus improving the thermal stability of the PSR-cellulose. These perspectives can be confirmed by the residual mass of the samples at the end of the thermal decomposition (Table 6). Hu [34] et al. concluded that the alkali bleaching treatment removed most of the lignin and reduced the strength of the lignin–cellulose complex, which made the lignin less thermally stable and showed a decreasing trend in the residual amount of thermal decomposition. At 500 °C, the cellulose had been completely decomposed, and the residual mass of PSR-cellulose was 20%, and the residual mass of CNCs by ZrP co-catalyzed hydrolysis was 19%. Compared to PSR-cellulose, CNCs are less thermally stable and have a reduced residual mass due to the absence of the lignin–cellulose complex in nanocellulose. On the other hand, the thermal stability of CNCs is reduced owing to the introduction of phosphate groups into the crystalline region of the cellulose during the preparation of CNCs.

**Figure 6:**
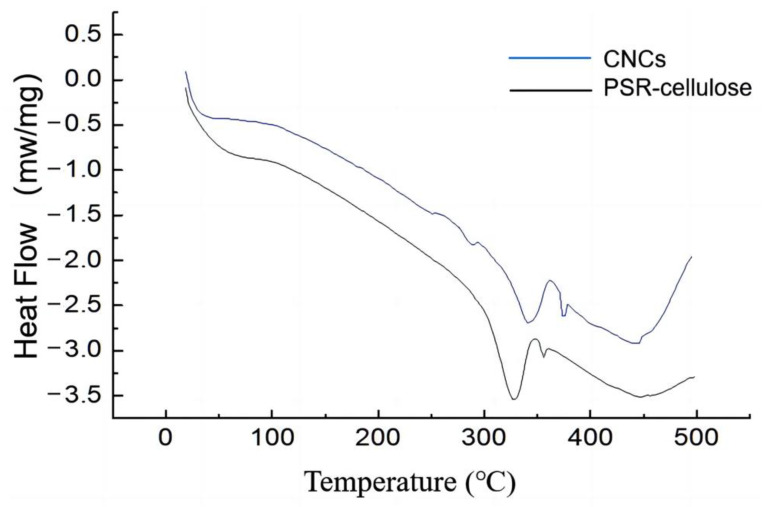
DSC profiles of PSR-Cellulose and CNCs.

Figure 6 shows the DSC profiles of PSR-Cellulose and CNCs. Both samples have two distinct intervals of heat uptake changes in DSC curves. The initial heat uptake process occurred before 120 °C, representing the moisture loss due to evaporation, which corresponds to the analysis of TGA. The evaporation process in PSR occurs in a wider temperature interval, mainly because of the presence of the lignin–cellulose complex, which enhances the sorption of water by the PSR and makes it more favorable for water retention. While CNCs have only a single adsorption force on the water, which leads to the water loss process occurring in a narrow temperature interval. The second heat absorption process is the thermal decomposition process of cellulose. During the CNCs preparation process, the hydrolysis of the glycosidic bonds breaks the long molecular chains of cellulose, resulting in a lower onset decomposition temperature for CNCs than for PSR-cellulose. The enthalpy values resulting from the evaporation of water and thermal decomposition processes in the PSR-cellulose are higher than those of CNCs (Table 7), which can be attributed to the strengthening of hydrogen bonds by the lignin–cellulose complex present in the PSR-cellulose.

## 4. Conclusions

This study explored the feasibility of ZrP-assisted phosphoric acid co-catalyzed hydrolysis PSR for the enhanced extraction of CNCs. The treatment conditions (the phosphate acid concentration, reaction temperature, and reaction time) were optimized by a quadratic model from RSM-BBD. The CNCs yield increased with an increase in the phosphate concentration, reaction temperature, and reaction time when the three independent variables were at low levels. When each of the three conditions reached an inflection point, the yield decreased as they increased. The optimum conditions were found at 49% phosphate acid, 65 °C, and a 5 h reaction time, resulting in a CNCs yield of 50.00%, which was closely coherent with the predicted value of 49.27%. The characterization analyses confirmed that ZrP could effectively assist the phosphate acid hydrolysis of PSR for the preparation of CNCs with a smaller crystallite size, higher crystallinity, and stable dispersion in an aqueous medium. This work has a significant reduction in the phosphoric acid consumption, process safety, low corrosiveness, high economic efficiency, and high yield of the CNCs obtained. These findings may contribute to the development of practical processes for the high-volume extraction of CNCs from PSR and potentially other biomasses.

## Figures and Tables

**Table 1:** Levels of the factors chosen for the trials.

Factors	Level		
	−1	0	+1
Phosphate acid concentration A (%)	45	50	55
Reaction temperature B (°C)	60	65	70
Reaction time C (h)	4	5	6

**Table 2:** The content of major composition in PSR.

Growth Cycle	Protein Content (%)	Fiber Content (%)	Fat Content (%)	Nitrogen Free Extract (%)	Ash Content (%)
4 week	10.8	28.5	3.8	43	13.9
6 week	8.8	32.2	3.5	42.6	12.9
8 week	8.7	32.8	3.3	44.3	10.9
10 week	6.5	33	2.7	46.4	11.4
12 week	5.9	31.9	2.9	49	10.3

**Table 3:** Experimental designs and results.

Run	Model Parameters			Responses
	A (%)	B (°C)	C (h)	Yield (%)
1	−1 (45)	−1 (60)	0 (5)	42.14
2	1 (45)	−1 (60)	0 (5)	40.00
3	−1 (45)	1 (70)	0 (5)	42.85
4	1 (55)	1 (70)	0 (5)	41.04
5	−1 (45)	0 (65)	−1 (4)	44.36
6	1 (55)	0 (65)	−1 (4)	42.29
7	−1 (45)	0 (65)	1 (6)	43.40
8	1 (55)	0 (65)	1 (6)	41.00
9	0 (50)	−1 (60)	−1 (4)	43.56
10	0 (50)	−1 (60)	−1 (4)	44.64
11	0 (50)	1 (70)	1 (6)	41.70
12	0 (50)	1 (70)	1 (6)	42.82
13	0 (50)	0 (65)	0 (5)	47.69
14	0 (50)	0 (65)	0 (5)	47.70
15	0 (50)	0 (65)	0 (5)	48.44
16	0 (50)	0 (65)	0 (5)	48.00
17	0 (50)	0 (65)	0 (5)	49.00

**Table 4:** ANOVA for Response Surface Quadratic Model.

Source	Sum of Squares	df	Mean Square	F-Value	Prob > F
Model	135.22	9	15.02	67.60	<0.0001
A	8.85	1	8.85	39.84	0.0004
B	1.95	1	1.95	8.76	0.0211
C	4.42	1	4.42	19.86	0.0029
AB	0.027	1	0.027	0.12	0.7359
AC	0.029	1	0.029	0.13	0.7297
BC	3.240 × 10^−4^	1	3.240 × 10^−4^	1.458 × 10^−3^	0.9706
A^2^	52.62	1	52.62	236.72	<0.0001
B^2^	41.01	1	41.01	184.50	<0.0001
C^2^	14.65	1	14.65	65.92	<0.0001
Residual	1.56	7	0.22		
Lack of Fit	0.31	3	0.10	0.33	0.8039
R^2^ = 0.9886; Adj R^2^ = 0.9740.					

**Table 5:** Zeta potential values of CNCs prepared by ZrP co-catalyzed hydrolysis.

Sample	Zeta Potential (mV)
PSR cellulose	−5.4
45% PA, 65 °C, 5 h	−19.4
50% PA, 65 °C, 5 h	−24.5
55% PA, 65 °C, 5 h	−21.3
50% PA, 60 °C, 5 h	−15.8
50% PA, 70 °C, 5 h	−18.7
50% PA, 65 °C, 4 h	−23.9
50% PA, 65 °C, 6 h	−17.3

**Table 6:** Thermal behavior of PSR-cellulose and CNCs.

Sample	Oneset Temperature (°C)	Maximum Temperature (°C)	Char Residue (wt.%)
PSR-cellulose	321.8	351.0	20
CNCs	313.5	330.4	19

**Table 7:** Thermal parameters of Pennisetum Sinese Roxb and CNCs obtained from DSC analysis.

Sample	Initial Temperature (°C)	Peak Temperature (°C)	Enthalpy of Pyrolysis ΔH (J/g)
PSR-cellulose	21.0 282.9 305.6 374.4	50.0 (endo) 297.9 (endo) 344.2 (endo) 379.0 (exo)	115.0 34.85 339.0 75.0
CNCs	24.4 296.8 377.0	54.0 (endo) 344.2 (endo) 380.3 (exo)	99.0 209.3 73.7

## Data Availability

The data presented in this study are available on request from the corresponding author.
